# Superconductivity-induced re-entrance of the orthorhombic distortion in Ba_1−*x*_K_*x*_Fe_2_As_2_

**DOI:** 10.1038/ncomms8911

**Published:** 2015-07-31

**Authors:** A. E. Böhmer, F. Hardy, L. Wang, T. Wolf, P. Schweiss, C. Meingast

**Affiliations:** 1 Institut für Festkörperphysik, Karlsruhe Institute of Technology, 76021 Karlsruhe, Germany

## Abstract

Detailed knowledge of the phase diagram and the nature of the competing magnetic and superconducting phases is imperative for a deeper understanding of the physics of iron-based superconductivity. Magnetism in the iron-based superconductors is usually a stripe-type spin-density-wave, which breaks the tetragonal symmetry of the lattice, and is known to compete strongly with superconductivity. Recently, it was found that in some systems an additional spin-density-wave transition occurs, which restores this tetragonal symmetry, however, its interaction with superconductivity remains unclear. Here, using thermodynamic measurements on Ba_1−*x*_K_*x*_Fe_2_As_2_ single crystals, we show that the spin-density-wave phase of tetragonal symmetry competes much stronger with superconductivity than the stripe-type spin-density-wave phase, which results in a novel re-entrance of the latter at or slightly below the superconducting transition.

## Abstract (web summary)

The interplay between magnetic and superconducting phases is important to understand the physics of iron-based superconductivity. Here, the authors use thermodynamic measurements on Ba_1−*x*_K_*x*_Fe_2_As_2_ single crystals to provide details of its phase diagram and the re-entrance of a *C*_2_ spin-density-wave phase.

## Graphical abstract

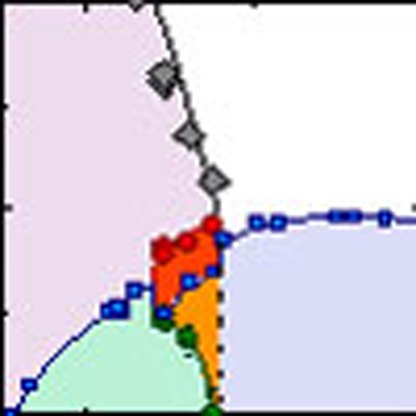

Unconventional superconductivity often arises around the point where some kind of magnetic order (either antiferromagnetic or ferromagnetic) is suppressed by a tuning parameter, such as pressure or chemical substitution^1^,^2^,^3^. This led to the idea that superconducting pairing in these materials may result from the low-energy magnetic fluctuations surrounding the quantum critical point where magnetism is suppressed to zero temperature^4^. The magnetic ground state of typical iron-based superconducting parent compounds is a stripe-type antiferromagnetic spin-density wave (SDW) phase, which breaks the *C*_4_ symmetry of the high-temperature tetragonal paramagnetic phase and is closely related to a tetragonal-to-orthorhombic structural distortion of the lattice^5^. An important debate, triggered by the observation that this orthorhombic structural distortion sometimes occurs slightly above the magnetic transition^6^, concerns the respective roles of spin and orbital degrees of freedom^7^,^8^. In the orbital picture, the structural transition is due to orbital ordering and can trigger a, secondary, magnetic transition^8^. In the so-called spin-nematic scenario, on the other hand, magnetism is essential and the structural transition is really just the first step of the magnetic transition, induced by an Ising-nematic degree of freedom associated with the emerging stripe-type magnetic order^9^. Importantly, both orbital and spin fluctuations are candidates for the superconducting pairing^7^,^8^.

A surprising recent result is the observation of a *C*_4_-symmetric tetragonal magnetically ordered phase in Ba_1−*x*_Na_*x*_Fe_2_As_2_ using neutron scattering^10^, which was taken as evidence for the spin scenario because the existence of such a phase can hardly be reconciled with orbital order being a prerequisite for magnetism^10^. Up to now, a similar *C*_4_-magnetic phase has not been observed in the closely related Ba_1−*x*_K_*x*_Fe_2_As_2_ system, which was the first iron-based superconductor of 122-type stoichiometry to be discovered^11^ and has the highest *T*_*c*_=38 K within this family. However, an unidentified phase transition in resistivity measurements under pressure^12^ hints that an additional instability is close by. Earlier theoretical work indicates the close proximity in energy between *C*_4_- and *C*_2_-symmetric magnetic structures^13^.

Here we re-examine the phase diagram of Ba_1−*x*_K_*x*_Fe_2_As_2_ in unprecedented detail using thermal-expansion and specific-heat measurements of very high-quality single crystals. Within a narrow composition range, we observe a *C*_4_-symmetric magnetic phase, which apparently has been missed in previous investigations^12^,^14^,^15^,^16^. In strong contrast to Na-doped BaFe_2_As_2_, the *C*_4_-phase is, however, not stable to zero temperature and reverts back to the *C*_2_ phase in the vicinity of the onset of superconductivity. This preference of superconductivity for the stripe-type *C*_2_- over the *C*_4_-magnetic state in this system may provide important clues about the superconducting pairing mechanism^17^.

## Results

### Orthorhombic distortion

**Figure 1:**
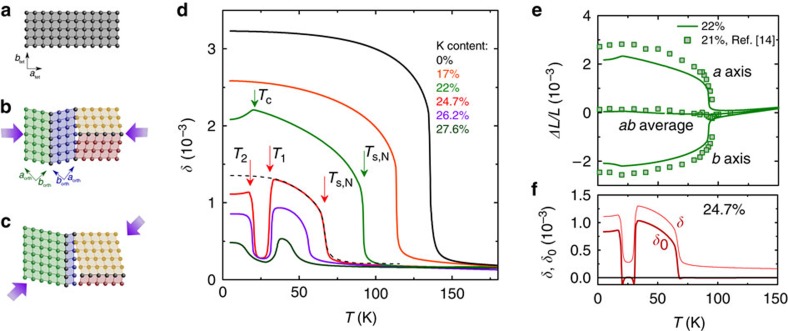
Orthorhombic distortion of Ba_1−*x*_K_*x*_Fe_2_As_2_ as measured using a capacitance dilatometer. (**a**) Schematic representation of the tetragonal *C*_4_ high-temperature phase. (**b**) Representation of the structural domains formed in the orthorhombic *C*_2_ phase (‘twins', indicated by different colours). The domain structure is unaffected by the force from the spring-loaded dilatometer if it is applied along the tetragonal [100] in-plane direction (purple arrows). (**c**) Representation of the mostly ‘detwinned' state, achieved by applying the dilatometer force along [110], which selects the domains with their (shorter) orthorhombic *b* axis along the direction of the applied force. (**d**) Temperature dependence of the orthorhombic distortion *δ*=(*a*−*b*)/(*a*+*b*) of underdoped Ba_1−*x*_K_*x*_Fe_2_As_2_ obtained using difference of ‘twinned' and ‘detwinned' data from our high-resolution capacitance dilatometer. Abrupt changes of *δ* mark phase transitions, examples of which are indicated by vertical arrows. (**e**) Good agreement between our results (continuous lines), and results from neutron powder diffraction^14^ (symbols) for the thermal expansion of the *a* and *b* axis demonstrates the reliability of our technique. (**f**) Orthorhombic distortion corrected for the effect of the applied force, *δ*_0_, for the sample with 24.7% K content, distinguishing tetragonal and orthorhombic phases (see Supplementary Figs 1 and 2 for details on the measurement of the orthorhombic distortion).

Figure 1 presents our first main result, the orthorhombic distortion *δ*=(*a*−*b*)/(*a*+*b*) (*a* and *b* are the in-plane lattice constants) versus temperature of underdoped Ba_1−*x*_K_*x*_Fe_2_As_2_ obtained using our high-resolution capacitance dilatometer^18^. Even though dilatometry is a macroscopic probe, the thermal expansion of the individual *a* and *b* axes, as well as the distortion *δ*, can be derived using the difference between ‘twinned' and ‘detwinned' data sets, as shown previously for FeSe (ref. 19) and detailed in the supplementary material (Supplementary Figs 1 and 2). The reliability of this method, which has higher resolution by several orders of magnitude than either neutron or X-ray diffraction experiments, is demonstrated by the close match to results of neutron powder diffraction^14^ (see Fig. 1e). In Ba_1−*x*_K_*x*_Fe_2_As_2_, the structural and magnetic transitions are coincident and first-order over the entire phase diagram^14^. Our curves in Fig. 1d, indeed, exhibit the well-known increase of *δ* at *T*_s,N_ and the subsequent suppression of *δ* below *T*_c_ (ref. 20) for 22% K content. In the small concentration range ∼24.7%–27.6% K content we find a surprising new result, namely, a sudden reduction of the orthorhombic distortion at *T*_1_<T_s,N_, followed by a sudden increase at *T*_2_ to a value slightly below its maximum value. This sudden reduction of *δ* strongly suggests that these Ba_1−*x*_K_*x*_Fe_2_As_2_ samples undergo a similar transition to a *C*_4_-magnetic phase as found in Ba_1−*x*_Na_*x*_Fe_2_As_2_ (refs 10, 21). The increase of *δ* at *T*_2_, on the other hand, is an indication for re-entrance of the *C*_2_-SDW phase. To prove that the intermediate phase is truly tetragonal, we have corrected the data of the 24.7% K crystal for the finite stress applied during the measurement, which induces non-zero distortion *δ* even in *C*_4_-symmetric phases (Fig. 1f, see Supplementary Figs 1 and 2). The corrected value, *δ*_0_, indeed vanishes completely (to within the extremely small value ∼0.01 × 10^−3^, related to our error bar) in the intermediate phase, as expected for a truly tetragonal *C*_4_ phase.

Similar to inverse melting^22^, the counterintuitive transition at *T*_1_ into a state of seemingly higher symmetry on lowering of the temperature does not violate the laws of thermodynamics. Rather, it convincingly demonstrates that the structural distortion is not the primary order parameter and that other degrees of freedom must be involved. The absolute measure for disorder is the entropy of the isolated system, which never increases with decreasing temperature. In the following we investigate the novel re-entrant transitions at *T*_1_ and *T*_2_ and their relation to superconductivity by examining the electronic entropy/heat capacity of the system. Moreover, the fact that *δ* below *T*_2_ is still reduced from the value expected by extrapolation from the *C*_2_-SDW phase (dashed black line for the 24.7% sample, red curve in Fig. 1d) suggests that the superconducting transition lies somewhere within the intermediate *C*_4_ phase. Since no clear signature of superconductivity can be observed in the *δ* (*T*) curves, heat-capacity data are also crucial to nail down the location of the bulk superconducting transition.

### Specific heat and electronic entropy

**Figure 2:**
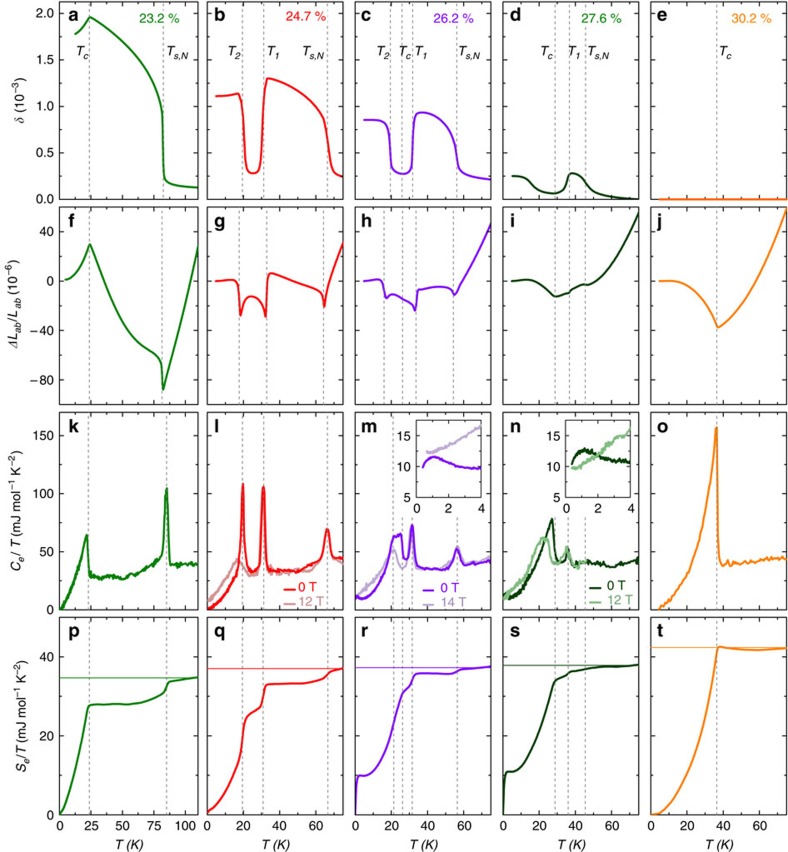
Phase transitions in thermal expansion and electronic specific heat for 5 samples of different K content. (**a**–**e**) Orthorhombic distortion *δ*, (**f**–**j**) average in-plane length change Δ*L*_*ab*_/*L*_*ab*_ for ‘twinned' samples (**k**–**o**) electronic specific heat *C*_e_/*T*, (**p**–**t**) electronic entropy divided by temperature *S*_e_/*T*. Horizontal lines indicate the value of *S*_e_/*T* of the high-temperature paramagnetic phase which is lost on cooling through the various transitions. (**l**–**n**) also show data measured in a large magnetic field and the insets show a magnification of the low-temperature region. Vertical lines mark the transitions temperatures labelled in panels (**a**–**e**).

Figure 2 presents our electronic heat capacity *C*_e_/*T* and electronic entropy divided by temperature 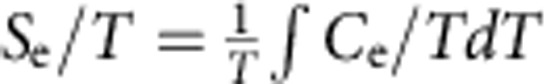, complemented by *δ*, and the temperature-dependent in-plane length change Δ*L*_*ab*_/*L*_*ab*_ for ‘twinned' samples (*c* axis data and thermal-expansion coefficients are given in the Supplementary Fig. 3) for various compositions. In a Fermi liquid, *S*_e_/*T* is expected to be constant, and how the entropy is reduced on cooling through the transitions provides important information about the strength of the competing orders. For the 23.2% sample, the usual first-order tetragonal-to-orthorhombic transition occurs at *T*_s,N_=81 K and results in a sizable reduction of *S*_e_/*T*. The remaining *S*_e_/*T* is lost through superconductivity with an onset at *T*_c_=24 K (see Fig. 2a,f,k,p).

The lowest K concentration for which the *C*_4_-magnetic phase is observed is 24.7% (Fig. 2b,g,l,q). Surprisingly, we do not observe a clear signature of a superconducting transition neither in *C*_e_/*T* nor in *L*_*ab*_ for this crystal, while strong first-order peaks indicate *T*_s,N_, *T*_1_ and *T*_2_ unambiguously. Nevertheless, the heat-capacity data show that the Fermi-surface is completely gapped at low temperature, implying a superconducting ground state. *T*_c_ is located by applying a large magnetic field. Notably, in a magnetic field of 12 T, the sharp peak at *T*_2_ disappears and is replaced by a broadened step-like anomaly, which is slightly shifted downward in temperature from the peak at *T*_2_, while the transitions at *T*_1_ and *T*_s,N_ are hardly affected. The step-like anomaly, the shift and the broadening in field are expected for a superconducting transition, and we therefore identify this transition with *T*_c_. This result implies that the strong specific-heat peak in zero-field at *T*_2_ is a novel combined structural, magnetic and superconducting first-order transition, in which a re-entrance of the *C*_2_-SDW phase occurs. How exactly a magnetic field tunes the system from a first- to second-order transition at *T*_*c*_ is a direction for future studies.

Increasing the K-content by just over 1% (to 26.2% K) drastically changes the behaviour. Superconductivity at *T*_c_=26 K can now easily be identified by the clear second-order specific-heat anomaly and a small kink in *L*_*ab*_ (Fig. 2h,m). The specific-heat anomaly is again broadened and shifted to lower temperatures by a high magnetic field. Superconductivity now coexists with the intermediate *C*_4_ phase and reentrance of the *C*_2_ phase occurs at *T*_2_<*T*_c_, as evidenced by the increase of *δ* and *L*_*ab*_ (Fig. 2c,h). Surprisingly, there is only a very small anomaly in the heat capacity at *T*_2_ (Fig. 2m). At still a slightly higher K content (27.6% K, Fig. 2d,i,n,s), no more anomaly that could be associated with *T*_2_ is observed in either the heat capacity or in *L*_*ab*_. The weak re-emergence of the orthorhombic distortion at low temperature is likely induced by the stress applied for detwinning. For this sample, the reduction of *S*_e_/*T* is mainly due to superconductivity, and the magnetic and structural phase transitions at *T*_s,N_ and *T*_1_ play only a very minor role. Finally, Fig. 2e,j,o,t show the results for a sample with 30% K content, which undergoes only a superconducting transition. Note the larger specific-heat anomaly, which implies a considerably larger superconducting condensation energy than for the other samples.

Our heat-capacity data also show a striking low-temperature contribution to the electronic specific heat, or equivalently to the entropy, in the superconducting state for samples with 26.2 and 27.6% K content (see Fig. 2m,n,r,s). This feature, which is very reminiscent of the very small superconducting gaps found in KFe_2_As_2_ (ref. 23), is a sign of excited quasiparticles far below *T*_c_ and seems to occur only when the structural-magnetic transitions are weak, that is, induce only a small entropy change. From the position of the maximum in *C*_e_/*T*, we estimate for the size of the smallest superconducting gap Δ_SC_∼0.07*k*_B_*T*_c_ in the multigap system, which is even smaller than the ‘lilliputian' gaps in KFe_2_As_2_ (ref. 23). The occurrence of such an extremely small gap may be related to peculiar features of the Fermi surface resulting from a reconstruction at *T*_s,N_ and *T*_1_ (see below). When this reconstruction becomes weaker on K doping, some parts of the reconstructed Fermi surface may move to within the superconducting gap Δ_SC_ of the Fermi level and can contribute to the superconducting condensate, as recently argued by Koshelev *et al*.^24^. This would explain why this low-temperature feature suddenly disappears once *T*_s,N_ and *T*_1_ are suppressed by doping (see Fig. 2o,t).

### Phase diagram

**Figure 3:**
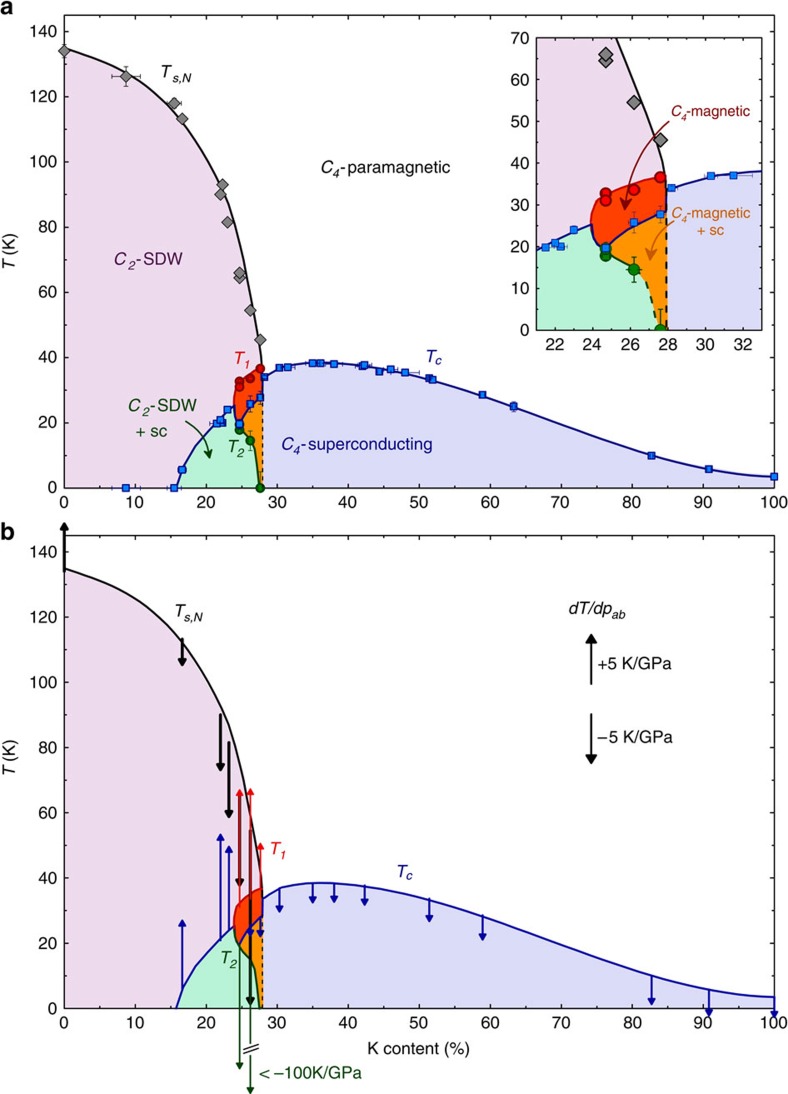
Detailed phase diagram of Ba_1−*x*_K_*x*_Fe_2_As_2_. (**a**) Phase diagram of Ba_1−*x*_K_*x*_Fe_2_As_2_ spanning the whole substition range. *T*_s,N_, *T*_1_, *T*_2_ and *T*_c_ (determined from the data shown in Fig. 2 and additional thermodynamic data) are shown by symbols. Lines and coloured areas are a guide to the eye indicating the five distinct thermodynamically ordered phases. A narrow region of a *C*_4_-symmetric (tetragonal) magnetic phase, occurring within the usual *C*_2_-symmetric (orthorhombic) SDW phase, is observed. It coexists with superconductivity with a reduced *T*_c_. Superconductivity-induced reentrance of the *C*_2_-SDW phase occurs at *T*_2_. The inset shows an enlarged view of the region containing the *C*_4_-magnetic phase. Vertical error bars indicate the uncertainty in the transition temperatures and horizontal error bars indicate the statistical error in the sample composition. (**b**) Predicted effect of uniaxial in-plane pressure *p*_*ab*_ on the phase diagram, from thermodynamic relations using our thermal-expansion and heat-capacity data. The direction of the arrows indicates the sign of the pressure derivative of the transition temperature and the length is proportional to its absolute value (see scale). Lines are reproduced from panel (**a**).

The transition temperatures from Fig. 2 are summarized in the phase diagram of Fig. 3a together with additional thermodynamic data covering the whole phase diagram^25^, (F. Hardy *et al*., manuscript in preparation). We find five distinct thermodynamic ordered phases, which all compete for the electronic entropy provided by the high-temperature *C*_4_-paramagnetic phase: *C*_2_ SDW, *C*_4_ magnetic, *C*_2_ SDW coexisting with superconductivity, *C*_4_ magnetic coexisting with superconductivity and *C*_4_-superconducting. Strikingly, *T*_c_ drops by about 7 K on going from the *C*_2_- to the *C*_4_-magnetic phase and, similarly, *T*_c_ increases by about 6 K at the boundary from the *C*_4_-magnetic to the *C*_4_-paramagnetic state. This points to a much stronger competition between superconductivity and the *C*_4_-magnetic phase than between superconductivity and the *C*_2_-magnetic phase, which is probably due to the additional pronounced suppression of entropy at *T*_1_ (see Fig. 2q). In particular, it would be clearly thermodynamically advantageous for superconductivity if the system would revert back to the *C*_2_-magnetic phase with the higher electronic entropy available for superconducting pairing. The system apparently does just this via a peculiar first-order magnetic, structural and superconducting transition at *T*_2_ for the crystal with 24.7% K content. On further K doping, *T*_1_ increases slightly, even though the entropy reduction at *T*_1_ becomes significantly weaker. Apparently, this renders the coexistence of superconductivity and the *C*_4_-magnetic phase possible, diminishing the driving force of the re-entrant transition at *T*_2_.

The interplay between different thermodynamic phases is also reflected in the effect of pressure on the phase diagram. We infer the effect of uniaxial, in-plane average, pressure *p*_*ab*_ on the phase diagram from our thermal-expansion and specific-heat data using thermodynamic relations (see Methods) and the results are shown in Fig. 3b. For example, when superconductivity coexists with the *C*_2_-SDW phase the uniaxial-pressure dependences *dT*_s,N_/*dp*_*ab*_<0 and *dT*_c_/*dp*_*ab*_>0 are of opposite sign and rather large showing the well-known competition of these two phases. Interestingly, a similar competition between the two magnetic phases is implied by the derivatives *dT*_s,N_/*dp*_*ab*_<0 and *dT*_1_/*dp*_*ab*_>0. However, *dT*_c_/*dp*_*ab*_≈−1 K GPa^−1^ is relatively small within the *C*_4_ magnetic phase, even though *T*_1_ and *T*_2_ are very sensitive to *p*_*ab*_. Note that the strong competition between superconductivity and the *C*_2_-SDW phase manifests itself by a strong coupling of both order parameters to the orthorhombic distortion, and clearly such a coupling is not possible in a tetragonal state, which might explain this small value. Finally, it is interesting that, within the whole range from 28 to 100% K content, the *T*_c_ line is very smooth and *dT*_c_/*dp*_*ab*_≈−(2−3) K GPa^−1^ changes only weakly with doping. This suggests that the superconducting state does not change over the whole phase diagram and, in particular, does not undergo a symmetry change^26^.

## Discussion

The *C*_4_ state which we observe in K-doped BaFe_2_As_2_ is very reminiscent of the magnetic *C*_4_ phase observed in Na-doped BaFe_2_As_2_ (ref. 10), and is also probably closely related to the pressure-induced phase found in Ba_1−*x*_K_*x*_Fe_2_As_2_ (ref. 12) (see Supplementary Fig. 4). This suggests that the occurrence of the *C*_4_ phase is a universal property of the hole-doped 122-materials, which are expected to be 'cleaner' than the electron-doped ones. The most striking difference to Ba_1−*x*_Na_*x*_Fe_2_As_2_ is the sudden re-emergence of *δ*, that is, the re-entrance of the *C*_2_-SDW phase, below *T*_2_≤*T_c_* in Ba_1−*x*_K_*x*_Fe_2_As_2_. One explanation for this difference may be the lower stability of the *C*_4_ phase, indicated by considerably lower values of *T*_1_. It is possibly related to a chemical pressure effect, since our results show that the magnetic *C*_4_ phase in Ba_1−*x*_K_*x*_Fe_2_As_2_ is extremely sensitive to (uniaxial) pressure (Fig. 3b). In particular, in-plane pressure is expected to strongly increase the extent of the *C*_4_-magnetic phase, similar to hydrostatic pressure^12^.

Several authors^10^,^17^,^27^,^28^,^29^,^30^ have suggested that the detailed nature of the additional magnetic phase can hold important clues regarding the spin-orbital debate. As pointed out in ref. 10, the existence of a *C*_4_-symmetric magnetic phase shows that the orthorhombic distortion is not necessary for magnetism which strongly supports the spin-nematic scenario. Concerning the magnetic structure, such a phase is possible when there are two antiferromagnetic propagation vectors and the iron magnetic moments are either non-collinear or non-uniform^13^,^27^,^31^. On the other hand, magnetic neutron scattering results on Ba_1−*x*_Na_*x*_Fe_2_As_2_ single crystals point to a re-orientation of the magnetic moments from in-plane to *c* axis orientated as the main element of the transition at *T*_1_ and call for further study on whether the magnetic structure really has *C*_4_ symmetry. Incidentally, the observed spin-re-orientation demonstrates that spin-orbit coupling cannot be neglected, and more theoretical work explicitly including this coupling is needed. Using the specific heat data, we can address the nature of these phase transitions from a thermodynamic point of view. In particular, the large entropy jump at the *T*_1_ transition is not expected for a simple spin reorientation. Rather, our data imply that a significant change of the band structure occurs, which may be more in line with the emergence of a second SDW order parameter in the itinerant spin-nematic viewpoint^12^,^17^. Moreover, our high-resolution data demonstrate that the orthorhombic distortion vanishes in the second magnetic phase with an error bar of less than 1% of its maximum value, which strongly suggests that the phase is truly *C*_4_-symmetric and that spin re-orientation is not the only element of the transition. Our data also show that superconductivity has a significant impact and can tip the balance between the two magnetic states in favour of the *C*_2_-symmetric one. Very recent theoretical works based on three-band itinerant fermionic or five-band tight-binding approaches in fact find surprisingly good qualitative agreement with our experimental phase diagram, suggesting that the physics of the hole-doped materials can be understood fairly well within these frameworks^17^,^30^. In particular, the suppression of superconductivity within the *C*_4_ phase and the re-entrance back into the *C*_2_ phase is captured in the work of ref. 30.

In summary, a new and very detailed phase diagram of Ba_1−*x*_K_*x*_Fe_2_As_2_, revealing intriguing new interactions between superconductivity and magnetism, has been derived from thermodynamic measurements. In particular, we find evidence for a narrow region of a *C*_4_-symmetric magnetic phase which competes more strongly with superconductivity than the *C*_2_-symmetric SDW phase. This competition between the two magnetic phases and superconductivity for the electronic entropy of the system results in a novel re-entrance of the *C*_2_-symmetric phase either as a magneto-structural transition within the superconducting phase or as a peculiar first-order concomitant superconducting, structural and magnetic transition, both of which are exceptional for strongly correlated systems. The strong reduction of entropy associated with the second magnetic phase suggests it to be a *C*_4_-type SDW in an itinerant scenario, rather than a simple spin re-orientation transition, in good agreement with recent theoretical work^17^,^30^. There are, however, still some unresolved issues, including the exact magnetic structure and the role of orbitals in this *C*_4_ SDW phase. The existence of the *C*_4_ phase at ambient pressure in high-quality single crystals opens up the possibility for studies using a large variety of more microscopic probes, such as magnetic neutron scattering, nuclear magnetic resonance, and angle-resolved photoemission studies, which will hopefully unravel some of these mysteries. Finally, we note that our preliminary thermodynamic studies on the Ba_1−*x*_Na_*x*_Fe_2_As_2_ system show that this system is even more complicated than thought at present^10^, and a detailed comparison between the K- and Na-doped systems will be very useful (L. Wang *et al*., manuscript in preparation).

## Methods

### Sample preparation and characterization

High-quality Ba_1−*x*_K_*x*_Fe_2_As_2_ samples were grown by a self-flux technique in alumina crucibles sealed in iron cylinders using very slow cooling rates of 0.2–0.4 °C per h. They were *in situ* annealed by further slow cooling to room temperature. Single crystals with a mass of 1–3 mg were chosen for measurement and the composition of the five main samples of this article was determined by refinement of four-circle single-crystal X-ray diffraction patterns of a small piece of each crystal to be *x*=0.232(3), 0.247(2), 0.262(3), 0.276(2), 0.302(35). Good homogeneity is attested by the sharp thermodynamic transitions. The phase diagram in Fig. 3 is compiled using thermodynamic measurements of samples whose K content was determined by either single-crystal X-ray diffraction refinement or energy dispersive X-ray spectroscopy.

### Thermal-expansion and specific-heat measurements

Uniaxial thermal expansion was measured in a home-made capacitance dilatometer^18^ and specific heat in a Physical Property Measurement System from Quantum Design. The electronic specific heat was obtained by subtracting (F. Hardy *et al*., manuscript in preparation), from the raw data, a concentration-weighted sum of the lattice heat capacity of KFe_2_As_2_ and Ba(Fe_0.85_Co_0.15_)_2_As_2_ derived from refs 23 and 32.

### Determination of uniaxial pressure derivatives

Uniaxial-pressure derivatives were obtained from the Clausius–Clapeyron relation *dT*/*dp*_*ab*_=*V*_*m*_(Δ*L*_*ab*_/*L*_*ab*_)/Δ*S* for first-order phase transitions (*T*_s,N_, *T*_1_ and *T*_2_) and from the Ehrenfest relation, *dT*/*dp*_*ab*_=*V*_*m*_Δ*α*_*ab*_/Δ(*C*/*T*) for second-order phase transitions (*T*_c_). Here, *p*_*ab*_ is the average in-plane pressure, *α*_*ab*_=1/*L_ab_dL*_*ab*_/*dT* the thermal expansion coefficient and Δ*L*_*ab*_, Δ*S* and Δ*α*_*ab*_ are the discontinuities at the phase transition and *V*_*m*_=61.4 cm^3^ mol^−1^ is the molar volume, which hardly changes on K substitution.

## Additional information

**How to cite this article:** Böhmer, A.E. *et al*. Superconductivity-induced reentrance of the orthorhombic distortion in Ba_1−*x*_K_*x*_Fe_2_As_2_. *Nat. Commun.* 6:7911 doi: 10.1038/ncomms8911 (2015).

## Supplementary Material

Supplementary Information Supplementary Figures 1-4, Supplementary Discussion and Supplementary References
