# Supplementary material for: Superconductivity-induced re-entrance of the orthorhombic distortion in Ba1−xKxFe2As2
Source: Nat Commun. 2015 Jul 31;6:7911. doi: 10.1038/ncomms8911 (PMC4532874; doi:10.1038/ncomms8911)
Supplement: Supplementary Information — Supplementary Figures 1-4, Supplementary Discussion and Supplementary References [file ncomms8911-s1.pdf]

SUPPLEMENTARY FIGURES

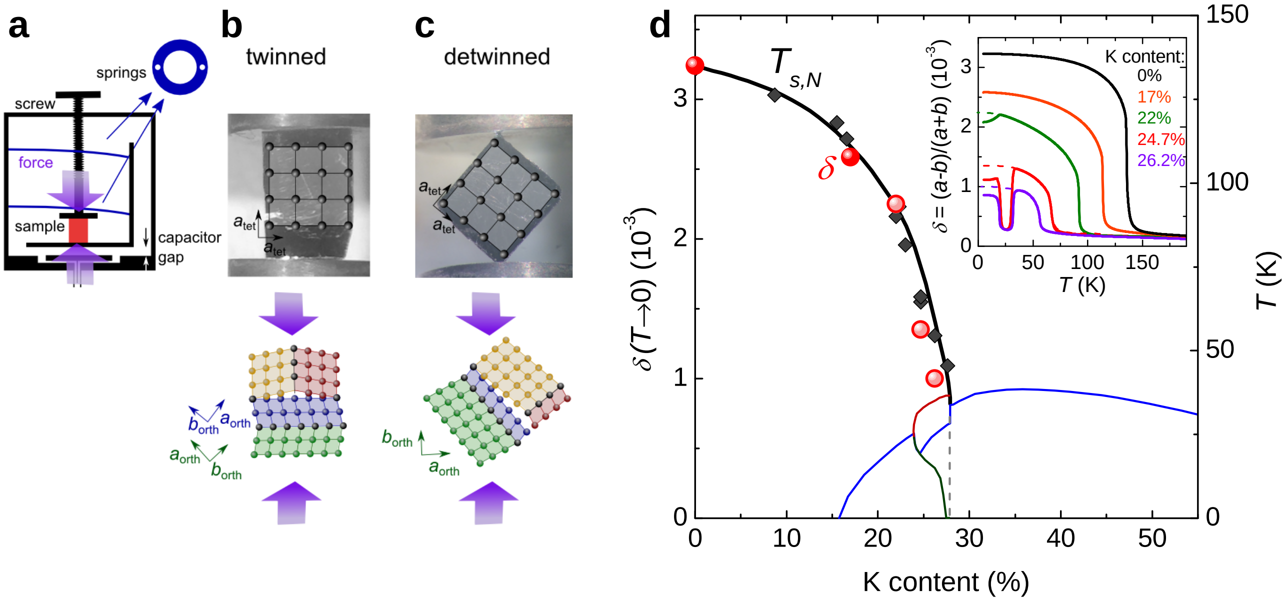

Supplementary Figure 1. Determination of the orthorhombic distortion in a capacitance dilatometer. (a) Scetch of the dilatometer with the applied uniaxial force indicated by the purple arrow. (b), (c) photographs of a sample inserted for 'twinned' and 'detwinned' measurement, respectively, with the tetragonal unit cell schematically indicated and a representation of the domain pattern in the orthorhombic state below. (d) Low-temperature value of the orthorhombic distortion $\delta(T \rightarrow 0)$ obtained from the data in Fig. 1 d of the main article (red circles, left scale). δ was extrapolated in cases when samples undergo additional phase transitions below T_s (open symbols). The inset shows this extrapolation. $T_{s,N}$ is also given and continous lines indicate the T_c, T_1 and T_2 (right scale, see Fig. 3 of the main article) .

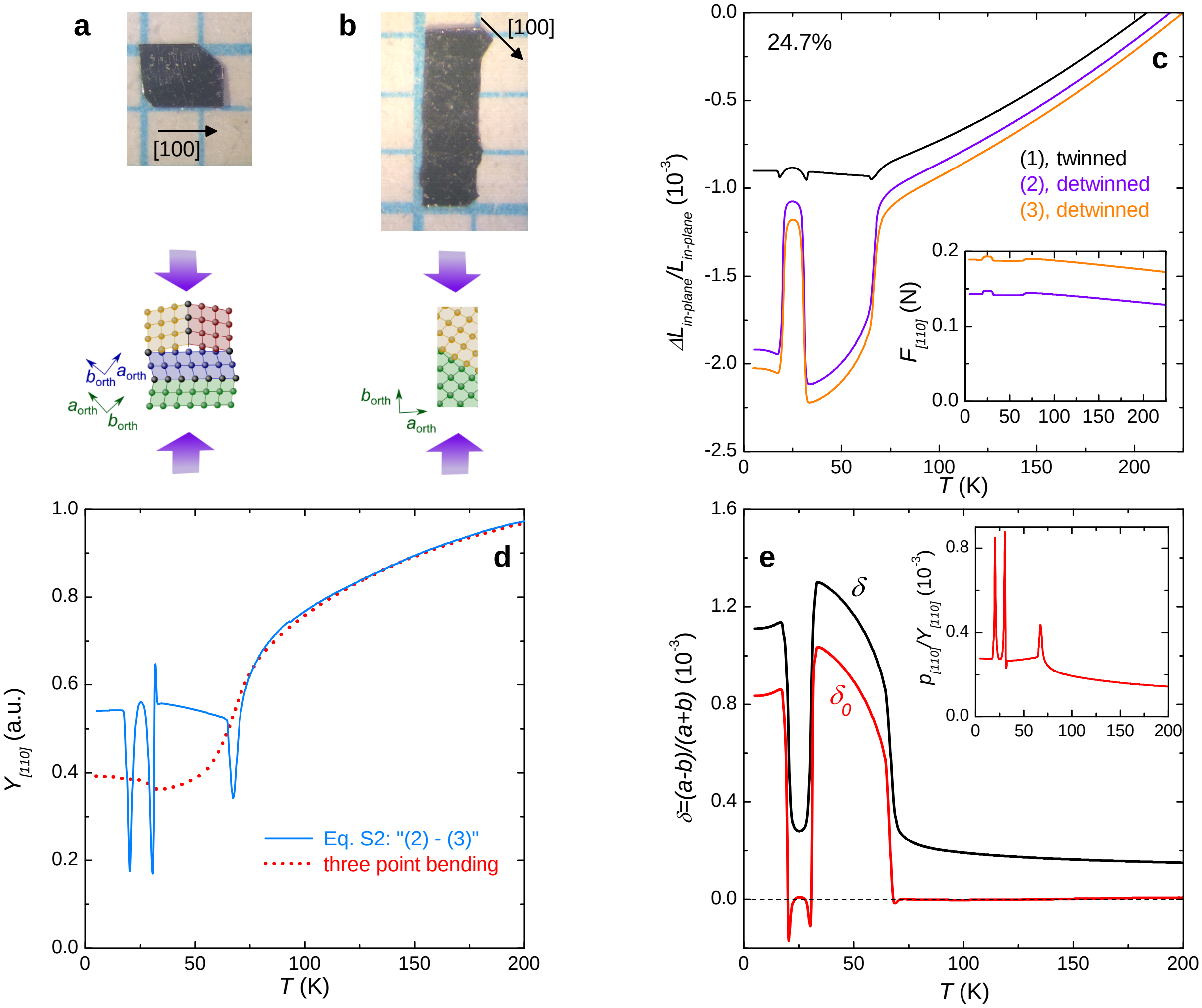

Supplementary Figure 2. Correction for the effect of applied force in the orthorhombic distortion using thermal expansion measurements under varying load in a capacitance dilatometer. Samples cut from the same larger crystal with 24.7% K content on a mm grid, (a) rectangle along [100] used for the twinned measurements, and (b) bar along [110], used for the detwinned measurements with controlled pressure. The tetragonal [100] direction and the domain pattern in the orthorhombic state are schematically indicated. (c) In-plane length change of the samples in (a) and (b) under the force $F_{[110]}$ applied by the spring-loaded dilatometer. In the 'twinned' measurement (1), (black curve) the force was applied along [100], hence $F_{[110]} = 0$. The values of $F_{[110]}$ are given in the inset. (d) Young's modulus $Y_{[110]}$ obtained from the data in (c) using equation 2. The dotted line shows the result of a three-point bending experiment in a capacitance dilatometer [1]. Curves in (c) were shifted for the $Y_{[110]}$ to match at high temperatures (see main text). (e) Orthorhombic distortion δ obtained by taking the difference between 'twinned' dataset (1) and 'detwinned' dataset (3) from panel (c) (black curve). The red curves shows δ_0, the distortion corrected for the effect of the applied force by subtracting the induced elastic length change $p_{[110]}/Y_{[110]}$ shown in the inset.

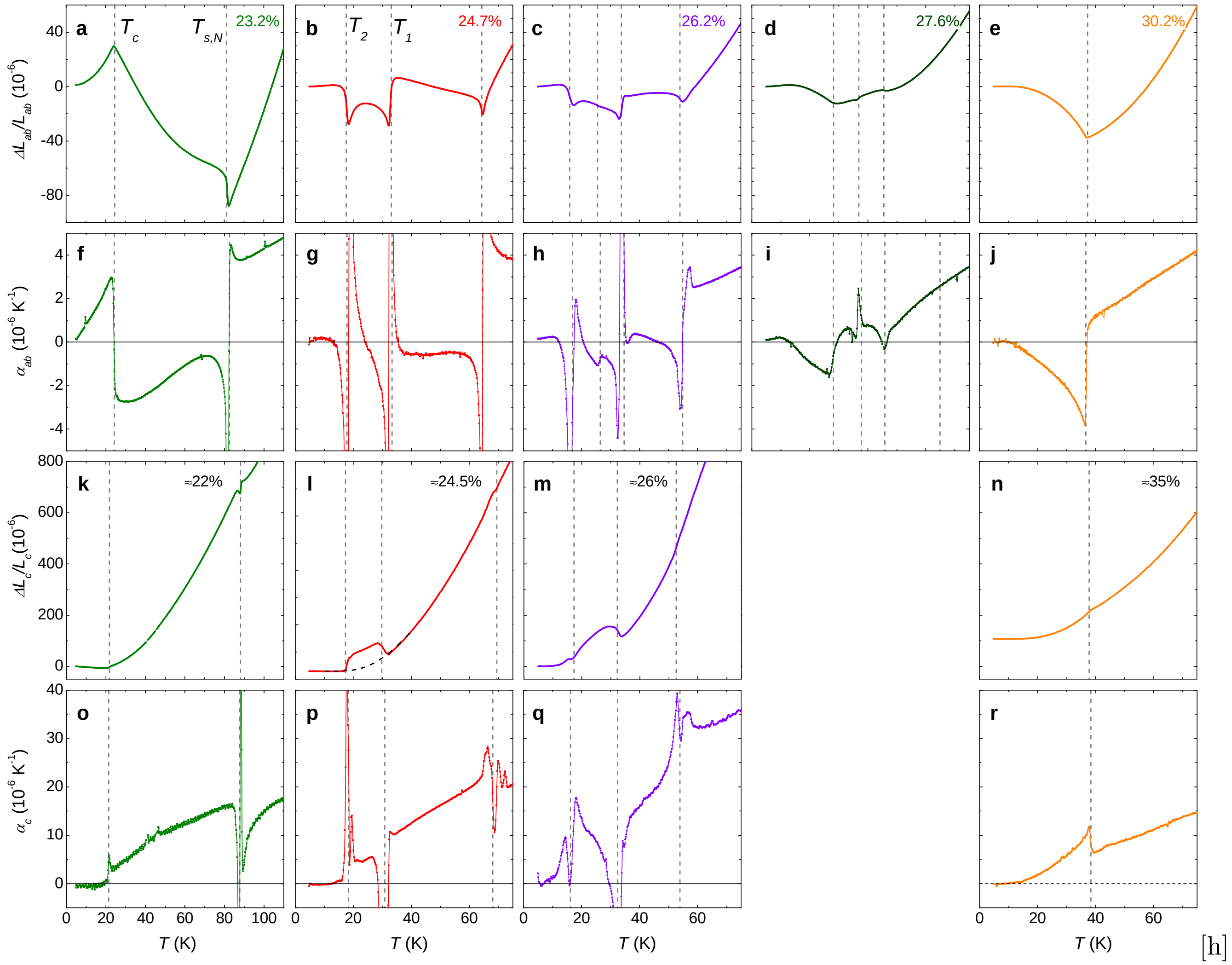

Supplementary Figure 3. Uniaxial thermal expansion of (Ba,K)Fe_2As_2 for $\sim 23\% - 30\%$ K content. (a-e) In-plane length change, as in Fig. 2 a-e of the main article. (f-j) Its temperature derivative, the uniaxial thermal-expansion coefficient α_{ab}, highlighting smaller anomalies at T_c. (k-n) c-axis length change of samples with similar composition. The thin line in (l) supports reentrance of the orthorhombic spin-density-wave phase at T_2. (o-r) The c-axis thermal-expansion coefficient α_c.

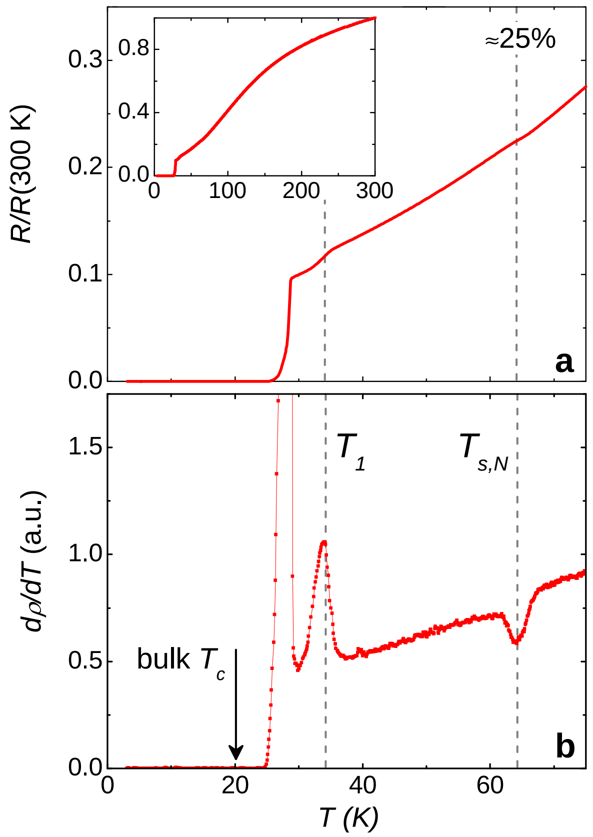

Supplementary Figure 4. Electrical resistance of a sample with ≈ 25% K content. (a) Electrical resistance normalized at room temperature. The inset shows the data over the whole temperature range. (b) Temperature derivative $d\rho/dT$ clearly showing sharp transitions at $T_{s,N}$ and T_1. The bulk T_c value as determined using thermodynamic measurements (indicated by the arrow) is significantly lower than the resistive T_c showing that it is not representative of the bulk.

SUPPLEMENTARY DISCUSSION

Measurements of the orthorhombic distortion

At the characteristic tetragonal-to-orthorhombic phase transition of the iron-based materials, the lattice expands along one diagonal of the tetragonal high-temperature unit cell (which then becomes the orthorhombic a axis) and shrinks along the other diagonal (subsequently the orthorhombic b axis). μm-sized structural domains, or 'twins', in which orthorhombic a and b axes are interchanged with respect to each other, are distributed approximately evenly within the sample [2], unless a symmetry-breaking uniaxial pressure (stress) is applied. Uniaxial pressure along the tetragonal [110] direction 'detwins' the sample such that, ideally, only one type of domains with the (shorter) orthorhombic b axis aligned along the direction of the applied pressure remains [3].

Our measurements of the orthorhombic distortion $\delta = (a - b)/(a + b)$ in the capacitance dilatometer [4] rely on the fact that the spring-loaded dilatometer cell intrinsically applies a small force F on the sample along direction of the measurement (see sketch in Supplementary Figure 1 a). This force is high enough to detwin the crystal when a sample is inserted such that it is directed along the tetragonal [110] direction and thus the thermal expansion of the orthorhombic b axis is obtained. In contrast, when the sample is inserted along the tetragonal [100] direction, the sample remains twinned and an average of orthorhombic a and b axis is measured. Typically, only samples aligned perfectly along [100] (Supplementary Figure 1b) remain 'well-twinned' and we insert the same sample along the "edges" (as shown in Supplementary Figure 1 c) to measure its b-axis expansion. The orthorhombic distortion can then be computed by taking the difference of the 'twinned' and the 'detwinned' data. The comparison with neutron powder diffraction data in Fig. 1 e of the main article demonstrates good agreement and supports the reliability of our technique. Supplementary Figure 1d shows the low-temperature value of the obtained orthorhombic distortion $\delta(T \rightarrow 0)$. An extrapolation is used when samples undergo another phase transition subsequently (see inset). The systematic evolution of $\delta(T \rightarrow 0)$ with K content and its good scaling with $T_{s,N}$ demonstrates reliable detwinning. Note that, even if a sample is not completely detwinned or, conversely, partly detwinned in the nominally 'twinned' measurement, δ will be underestimated but the temperature dependence will be affected only slightly.

It is important to realize that the applied force at all temperatures also induces an elastic change of the sample length, simply according to Hooke's law. This 'additional' length change is, hence, given by

$$\Delta L_{[110]}/L_{[110]} = S_{[110]}p_{[110]}, \quad (1)$$

where $\Delta L_{[110]}/L_{[110]}$ is the relative length change of the sample along [110], $p_{[110]}$ is the uniaxial pressure on the sample generated by the dilatometer force $F_{[110]}$ and $S_{[110]} = Y_{[110]}^{-1}$ is the sample's elastic compliance (inverse Young's modulus) along the tetragonal [110] direction. If $Y_{[110]}$ were temperature independent, the elastic effect would result in a constant contribution to δ. However, $Y_{[110]}$ of underdoped (Ba,K)Fe_2As_2 is strongly temperature dependent [1]. When a same sample is inserted along the "edges" (as shown in Supplementary Figure 1 c), this effect can, however, not be well quantified. In order to correct for the effect of the applied force, a bar of dimensions $(3.066 \times 1.08 \times 0.060)$ mm^3 with the longest dimension along [110] and the shortest dimension along the c axis has been cut from a larger sample with 24.7% K content (Supplementary Figure 2 b). The expansion along [110] of this bar-shaped piece has been measured under varying $F_{[110]}$ (measurements (2) and (3) in Supplementary Figure 2 a). The amount of applied force, which is varied by changing the initial gap of the capacitor, was determined accurately and is slightly temperature dependent (inset in Supplementary Figure 2d). An additional 'twinned' measurement (1) was conducted on another piece of the same larger crystal oriented along [100] (black curve in Supplementary Figure 2d). We assume that the two types of domains are evenly distributed in the 'twinned' measurement (1) and that the sample is completely detwinned in the measurements (2) and (3).

In principle, the Young's modulus can then be obtained by taking the difference of two measurements as

$$Y_{[110]} = \frac{p_{[110]}^{(n)} - p_{[110]}^{(m)}}{L_{[110]}^{(n)} - L_{[110]}^{(m)}}, \quad (2)$$

where $p_{[110]}$ is the applied uniaxial pressure and the superscripts (n) and (m) stand for measurements with different values of the applied force. A complication arises because $L_{[110]}^{(n)} - L_{[110]}^{(m)}$ is determined only up to a constant in the dilatometer. This constant offset can, however, be determined by shifting the curves in Supplementary Figure 2d vertically with respect to each other so that the Young's modulus obtained via eq. 2 matches the Young's modulus measured in a three-point bending experiment [1]. The advantage of using

eq. 2 over three-point bending is that $Y_{[110]}$ can be obtained over the whole temperature range. In contrast, the bending is strongly affected by the presence of structural twins [5], so that the 'intrinsic' monodomain behavior cannot be obtained in the orthorhombic state. Note that, in order to get agreement between the Young's moduli from the two measurement techniques, the curves (2) and (3) are shifted by 0.065×10^{-3} with respect to each other at $T = 150$ K, which corresponds to a reasonable value of $Y_{[110]}(150 \text{ K}) \approx 70$ GPa, when assuming that roughly 1/5 of the sample cross-section is in direct contact with the dilatometer cell. The so obtained $Y_{[110]}$ (Supplementary Figure 2e) exhibits the expected softening at the three structural phase transitions at $T_{s,N}$ T_1 and T_2 and is harder between these temperatures.

Using these results, we can, finally, quantify how the measured orthorhombic distortion is affected by the stress applied for detwinning. For example, in the above measurement, $F_{[110]} = 0.18$ N induces a distortion of $\delta = 0.16 \times 10^{-3}$ already at 150 K, i.e., far above the structural transition. The 'intrinsic' distortion corrected for the effect of applied stress, δ_0, is obtained by subtracting this elastically induced contribution $p_{[110]}/Y_{[110]}$ (inset in Supplementary Figure 2e). Indeed, we find that $\delta_0 = 0$ to within $\approx 0.01 \times 10^{-3}$ in both the tetragonal phases (Supplementary Figure 2e and Fig. 1 f), which shows that the procedure works well. Only in the immediate vicinity of the phase transitions, unphysical negative values of δ_0 are obtained, presumably because the linear stress-strain relationship of eq. 2 overestimates the effect of the applied stress in these regions.

Uniaxial thermal expansion

Supplementary Figure 3 shows additional thermal-expansion data for the samples in Fig. 2 of the main article and similar samples. The uniaxial in-plane thermal-expansion coefficient $\alpha_{ab} = d(\Delta L_{ab}/L_{ab})/dT$ is helpful to locate small anomalies. In particular, the small kink in L_{ab} at T_c of the 26.2% sample is clearly seen as a discontinuity in α_{ab} (see Supplementary Figure 3h). The size of the jump in α_{ab} at T_c is used to compute the pressure derivative of T_c shown in Fig. 3 of the main article. Supplementary Figure 3 k,l,m,n show the c-axis length changes $\Delta L_c/L_c$ for samples of similar composition. Note that, in order to get reliable data, "thicker" samples, i.e., samples which are longer along the c axis, had to be chosen and these often have a slightly more inhomogeneous K content, resulting in broadened anomalies.

Nevertheless, $T_{s,N}$, T_1 and T_2 can clearly be defined also in the c-axis data and the K content of these samples is estimated to be $\approx 24.5\%$ and $\approx 26\%$ K content from comparing $T_{s,N}$ with the phase diagram in Fig. 3 of the main article. In particular, an extrapolation of the $\Delta L_c/L_c$-data supports that re-entrance of the original C_2 SDW state occurs below T_2 (see dashed line in Supplementary Figure 3l). In general, the anomalies at the phase transitions in the c-axis data are smaller with respect to the "background" expansion , while they have opposite sign and similar shape with respect to the in-plane data. This seems to be a quite general feature of the iron-based superconductors [6, 7].

Resistivity

Supplementary Figure 4 shows the in-plane normalized electrical resistivity of a crystal with $\approx 25\%$ K content, as determined from $T_{s,N}$. The transitions at $T_{s,N}$ and T_1 are sharp and well-defined and closely match the thermodynamic data for samples of similar composition. Importantly, the transition at T_1 is very reminiscent of the unidentified transition at "T_0" observed in underdoped $Ba_{1-x}K_xFe_2As_2$ under pressure in Ref. 8, suggesting that the same phase transition is observed. The re-entrance of the C_2 SDW phase at T_2 within the superconducting state is, of course, impossible to observe using resistivity. We note that the drop in resistivity at T_c is quite broad and is significantly higher than the thermodynamically determined T_c value (indicated in the figure), showing that resistivity is not a good measure of the bulk superconducting transition.

Supplementary References

---

[1] Böhmer, A. E. *et al.* Nematic susceptibility of hole-doped and electron-doped $BaFe_2As_2$ iron-based superconductors from shear modulus measurements. *Phys. Rev. Lett.* **112**, 047001 (2014).

[2] Tanatar, M. A. *et al.* Direct imaging of the structural domains in the iron pnictides $A$$Fe_2As_2$ (A=Ca,Sr,Ba). *Phys. Rev. B* **79**, 180508 (2009).

[3] Fisher, I. R., Degiorgi, L. & Shen, Z. X. In-plane electronic anisotropy of underdoped '122' Fe-arsenide superconductors revealed by measurements of detwinned single crystals. *Reports*

on Progress in Physics **74**, 124506 (2011).

[4] Meingast, C. *et al.* Anisotropic pressure dependence of T_c in single crystal $YBa_2Cu_3O_7$ via thermal expansion. *Phys. Rev. B* **41**, 11299–11304 (1990).

[5] Schranz, W., Kabelka, H., Sarras, A. & Burock, M. Giant domain wall response of highly twinned ferroelastic materials. *Applied Physics Letters* **101**, 141913 (2012).

[6] Hardy, F., Adelmann, P., Wolf, T., v. Löhneysen, H. & Meingast, C. Large anisotropy uniaxial pressure dependencies of T_c in single crystalline $Ba(Fe_{0.92}Co_{0.08})_2As_2$. *Phys. Rev. Lett.* **102**, 187004 (2009).

[7] Meingast, C. *et al.* Thermal expansion and Grüneisen parameters of $Ba(Fe_{1-x}Co_x)_2As_2$ - a thermodynamic quest for quantum criticality. *Phys. Rev. Lett.* **108**, 177004 (2012).

[8] Hassinger, E. *et al.* Pressure-induced Fermi-surface reconstruction in the iron-arsenide superconductor $Ba_{1-x}K_xFe_2As_2$: Evidence of a phase transition inside the antiferromagnetic phase. *Phys. Rev. B* **86**, 140502 (2012).
